# Early detection of type 2 diabetes mellitus‐associated cognitive dysfunction: The potential of amide proton transfer‐weighted imaging

**DOI:** 10.1111/dom.70468

**Published:** 2026-02-02

**Authors:** Ju‐wei Shao, Ying Yang, Yun‐qian Zhang, Hong‐yan Dai, Jing Fang, Thomas C. Booth

**Affiliations:** ^1^ Department of Radiology The Affiliated Hospital of Yunnan University Kunming Yunnan China; ^2^ Department of Endocrinolgy The Affiliated Hospital of Yunnan University Kunming Yunnan China; ^3^ Department of Neurology The Affiliated Hospital of Yunnan University Kunming Yunnan China; ^4^ Institute for Health Sciences Kunming Medical University Kunming Yunnan China; ^5^ School of Biomedical Engineering & Imaging Sciences King's College London London UK

**Keywords:** amide proton transfer‐weighted, diabetes‐associated cognitive dysfunction, magnetic resonance imaging, mild cognitive impairment, type 2 diabetes mellitus

## Abstract

**Aims:**

Type 2 diabetes mellitus (T2DM) may lead to diabetes‐associated cognitive dysfunction (DACD). We aimed to develop an amide proton transfer‐weighted (APTw) magnetic resonance imaging (MRI) biomarker to assist early identification of T2DM with DACD.

**Materials and Methods:**

The study included 27 T2DM patients, comprising 19 with mild cognitive impairment (T2DM‐MCI) and 8 without (T2DM‐nMCI), along with 11 community‐based controls without MCI (CG‐nMCI). All participants completed neuropsychological tests and APTw‐MRI. We measured hippocampal APTw signal intensity (SI) in both groups, analysed its correlation with cognitive scores and assessed diagnostic performance using area under the curve (AUC).

**Results:**

In T2DM‐nMCI patients, left hippocampal head APTw SI showed a positive correlation with semantic verbal fluency (SVF; *r* = 0.770, *R* ^2^ = 0.593, *p* = 0.025) but a negative correlation with Wechsler Memory Scale‐Digit Span Test‐Backward (*r* = −0.802, *R* ^2^ = 0.643, *p* = 0.017). In T2DM‐MCI patients, left hippocampal head APTw SI positively correlated with SVF scores (*r* = 0.414, *R* ^2^ = 0.172, *p* = 0.044), while left tail values showed negative associations with Trail‐Making Test‐A (*r* = −0.333, *R* ^2^ = 0.111) and Auditory Verbal Learning Test‐Huashan version‐delayed recall (*r* = −0.376, *R* ^2^ = 0.141, both *p* ≤ 0.021). The left hippocampal body demonstrated diagnostic potential for T2DM‐nMCI (AUC = 0.793, *p* = 0.045), while the left hippocampal head showed higher discriminative power for T2DM‐MCI (AUC = 0.732, *p* = 0.034).

**Conclusions:**

APTw imaging suggested a spatially evolving pattern of hippocampal damage in T2DM, where the left body may show early alterations, with the left head potentially becoming more implicated upon MCI onset. These findings provide preliminary evidence supporting the potential of APTw as an early, non‐invasive biomarker for tracking neuropathological progression in DACD.

## 1 INTRODUCTION

Type 2 diabetes mellitus (T2DM) is a chronic metabolic disorder characterised by reduced insulin sensitivity and relative insulin deficiency.^1^ Its prevalence is steadily increasing, making it a significant global public health concern.^2^ Chronic hyperglycaemia can lead to chronic damage to, and dysfunction of, blood vessels and nerves; over time cerebral ischaemia and degenerative change can result in diabetes‐associated cognitive dysfunction (DACD).^3^, ^4^ DACD progresses through three stages: cognitive decline, mild cognitive impairment (MCI) and dementia.^5^ MCI is defined as a decline in one or more cognitive domains (typically less than 1–1.5 standard deviations [SD] from the mean), without impacting daily living, though it may eventually progress to dementia.^5^ Estimates of prevalence of co‐morbidity of MCI and T2DM patients appear to be around 45%.^6^ Detection of T2DM‐associated MCI is essential because MCI may be reversed with treatment, whereas once dementia is established, comorbid cases are mostly irreversible.^7^ However, the onset of cognitive dysfunction is often insidious, making early intervention challenging.^8^ Additional diagnostic challenges include cognitive assessments being confounded by education level leading to inaccurate results.^9^ While neuropsychological tests such as the Mini‐Mental State Examination (MMSE) and Montreal Cognitive Assessment (MoCA) serve as the clinical cornerstone for diagnosing MCI, they are subjective, performance‐based measures that can be influenced by socio‐demographic factors. Crucially, they primarily detect functional decline only after the underlying cognitive dysfunction has manifested. In contrast, amide proton transfer‐weighted (APTw) imaging has the potential to provide an objective, biological correlate of the underlying neuropathology. By detecting changes in mobile protein concentrations, it has the potential to identify early brain alterations even before they are fully expressed as cognitive symptoms, offering a complementary and potentially more sensitive diagnostic approach.

Recent studies suggested that T2DM shares pathophysiological similarities with Alzheimer disease (AD). The underlying neurobiological mechanisms are complex, involving multiple aspects such as impaired insulin signalling pathways, chronic inflammatory responses, oxidative stress and impaired cerebral vascular endothelial function.^10^, ^11^, ^12^ These processes interact significantly with core pathophysiological alterations in AD, such as β‐amyloid (Aβ) deposition and tau pathology,^10^ forming a ‘dual‐hit’ model that collectively accelerates neurodegenerative progression.^13^ These changes in the brain occur before cognitive dysfunction or dementia become clinically evident, possibly contributing to the development of DACD.^14^, ^15^, ^16^

Current protein detection methods in the human brain have limitations: single‐photon emission computed tomography (SPECT) and positron emission tomography (PET) imaging for amyloid‐beta and p‐tau require radiation exposure and costly radioactive tracers. While cerebrospinal fluid assays provide direct measurements, they involve invasive lumbar punctures with poor patient compliance. Plasma tests are less invasive but suffer from reduced specificity due to blood–brain barrier effects and multiple phosphorylation sites.^17^, ^18^ In comparison, APTw imaging is a non‐invasive magnetic resonance imaging (MRI) method that detects endogenous contrast through proton exchange in proteins and macromolecules, is less expensive, and can avoid radiation exposure.^19^, ^20^ The APT effect is usually evaluated using the asymmetric magnetisation transfer rate (MTR_asym_) at an offset of 3.5 ppm,^21^ which is highly reproducible.^22^ Studies have shown that APTw imaging can detect abnormal protein levels in the brains of AD patients, plausibly aiding early diagnosis of cognitive dysfunction.^23^

This exploratory study aimed to find out whether APTw imaging can detect early cognitive changes in T2DM patients and thus offer a non‐invasive biomarker for the diagnosis of DACD. If validated beyond this study, such a biomarker could plausibly help identify patients for proactive clinical treatment and prevent many patients from progressing to dementia.

## 2 MATERIALS AND METHODS

### 2.1 Participants

**FIGURE 1:**
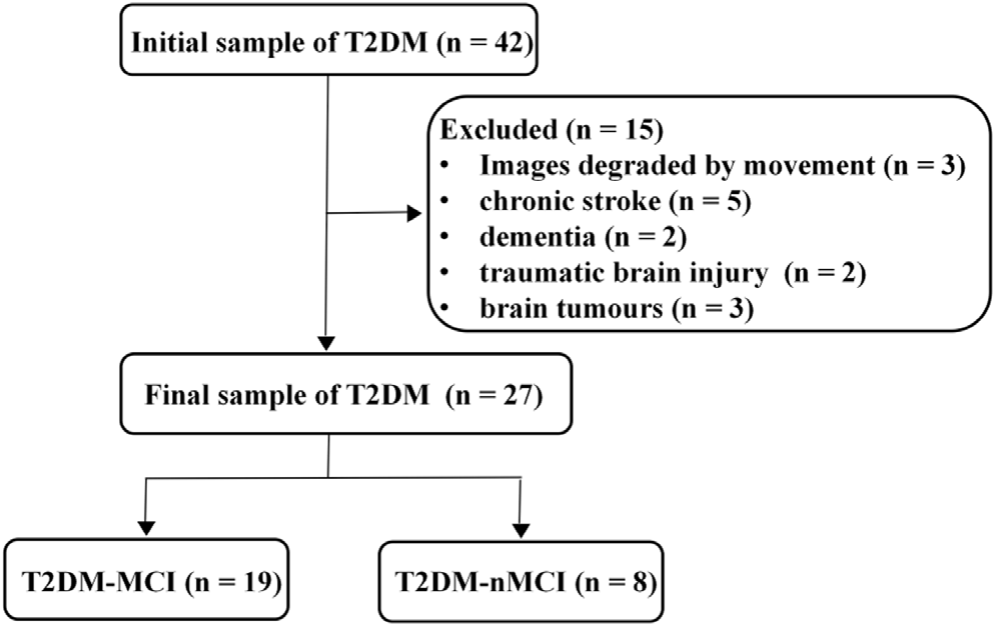
Flow diagram shows study inclusion and exclusion criteria. MCI, mild cognitive impairment; T2DM, type 2 diabetes mellitus; T2DM‐MCI group, T2DM with MCI; T2DM‐nMCI group, T2DM without MCI.

The study was approved by the Ethics Committee of The Affiliated Hospital of Yunnan University (202014), registered in the Chinese Clinical Trial Registry (registration number: CHICTR2000032446), conducted in accordance with the 1964 Declaration of Helsinki. We consecutively recruited 42 T2DM patients from the hospital and 20 non‐patient volunteers as controls from communities over a 6‐month period (a participant flowchart displaying the flow of T2DM patients included in analyses is presented in Figure 1).

The inclusion criteria for community‐based controls without MCI (CG‐nMCI) and T2DM groups were (1) age 50–70 years (2) right‐handedness (3) 6 years of schooling or beyond (elementary school graduation or beyond); (4) Hamilton Depression Rating Scale (HDRS‐17) scores <8 points; (5) ability to sign an informed consent form. Additional inclusion criteria for the T2DM group were (1) diagnosis according to the 1999 World Health Organization standard; (2) diagnosed with T2DM for at least 5 years. Additional inclusion criteria for the CG‐nMCI were (1) no diabetes mellitus; (2) no neurological disorder; (3) MoCA scale score ≧26; (4) MMSE scale score >24.

The exclusion criteria for both groups were (1) history of non‐T2DM conditions that may affect cognitive function including stroke, cerebral haemorrhage, neoplasms, craniocerebral trauma, hypothyroidism, vitamin deficiencies, anaemia, liver and kidney dysfunction and other diseases that can cause cognitive decline; (2) history of ocular disease; (3) language impairment or communication disorders; (4) contraindications to MRI; (5) alcohol dependence syndrome; (6) severe cardiopulmonary disease; (7) cancer; (8) moderate or severe small vessel disease as defined by age‐related white matter changes (ARWMC) rating scale score of >1.^25^

### 2.2 Cognitive assessment and group classification

The core concept of MCI aligns with the National Institute on Aging‐Alzheimer's Association (NIA‐AA) criteria^26^: (1) Concern regarding a change in cognition, (2) Impairment in one or more cognitive domains, (3) Preservation of independence in functional abilities, (4) Not demented. The assessment of these core criteria integrated participants' clinical symptoms with their cognitive performance. Cognitive function was evaluated in all participants using the MMSE and the MoCA. For the operational definition used in group classification, we applied the Peterson criteria^27^ to these cognitive scale scores. Patients were classified as MCI‐positive only if they displayed the specific profile of an MMSE score >24 (indicating generally preserved global cognition) concurrent with an MoCA score <26 (indicating measurable cognitive impairment). This approach ensured a clear distinction between groups. Patients who fulfilled only one of these two criteria were excluded from the MCI group to enhance the specificity of the diagnosis and to avoid the inclusion of individuals with ambiguous or borderline cognitive profiles (Table 1). Consequently, all individuals in the control group were confirmed as not having MCI (CG‐nMCI). The T2DM patients were categorised into two groups: the T2DM‐MCI group and the T2DM‐nMCI group.

**TABLE 1:** Operational criteria for participant group classification.

Participant group	Diagnostic criteria	MMSE score	MoCA score
CG‐nMCI	Control group without MCI	>24	≥26
T2DM‐nMCI	T2DM without MCI	>24	≥26
T2DM‐MCI	T2DM with MCI	>24	<26

Abbreviations: CG‐nMCI, control group without MCI; MCI, mild cognitive impairment; MoCA, Montreal Cognitive Assessment; MMSE, Mini‐Mental State Examination; T2DM, type 2 diabetes mellitus; T2DM‐MCI, T2DM with MCI; T2DM‐nMCI, T2DM without MCI.

In addition to the MoCA and MMSE, a battery of neuropsychological tests was administered to all participants by using different tools to assess multiple cognitive domains, including working memory (Wechsler Memory Scale‐Digit Span Test‐Total score/Forward/Backward [WMS‐DST‐T/F/B]), verbal working memory (Auditory Verbal Learning Test‐Huashan version [AVLT‐H]), attention and processing speed (Trail‐Making Test, Part A [TMT‐A]) and language function and executive function (semantic verbal fluency [SVF]). AVLT‐H includes AVLT‐H‐immediate recall (AVLT‐H‐I) and AVLT‐delayed recall (AVLT‐H‐D). We also used the HDRS‐17 to assess the presence of depression. Fasting blood‐glucose (FBG), glycosylated haemoglobin (HbA1c), triglyceride and total cholesterol levels were also tested.

### 2.3 Acquisition of imaging data

MRI examinations were performed with a 3 Tesla scanner (Philips Ingenia, Best, Netherlands) and an 8‐channel head phased array coil (Philips Ingenia, Best, Netherlands). T1‐weighted (T1WI) and T2‐weighted images (T2WI) were obtained for all patients. The entire hippocampus was scanned using the APT sequence. The scan parameters were as follows: repetition time (TR) = 5978 ms, echo time (TE) = 7.8 ms, gap = 0.0 mm, voxel size = 1.8 mm × 1.8 mm × 6 mm, number of excitation (NEX) = 1.00, field of view (FOV) = 230 mm × 180 mm, slice thickness = 6 mm, B1 (uT) = 2, saturation time = 2, 10 slices, scan time: 3 min 41 s.

### 2.4 Data analysis

**FIGURE 2:**
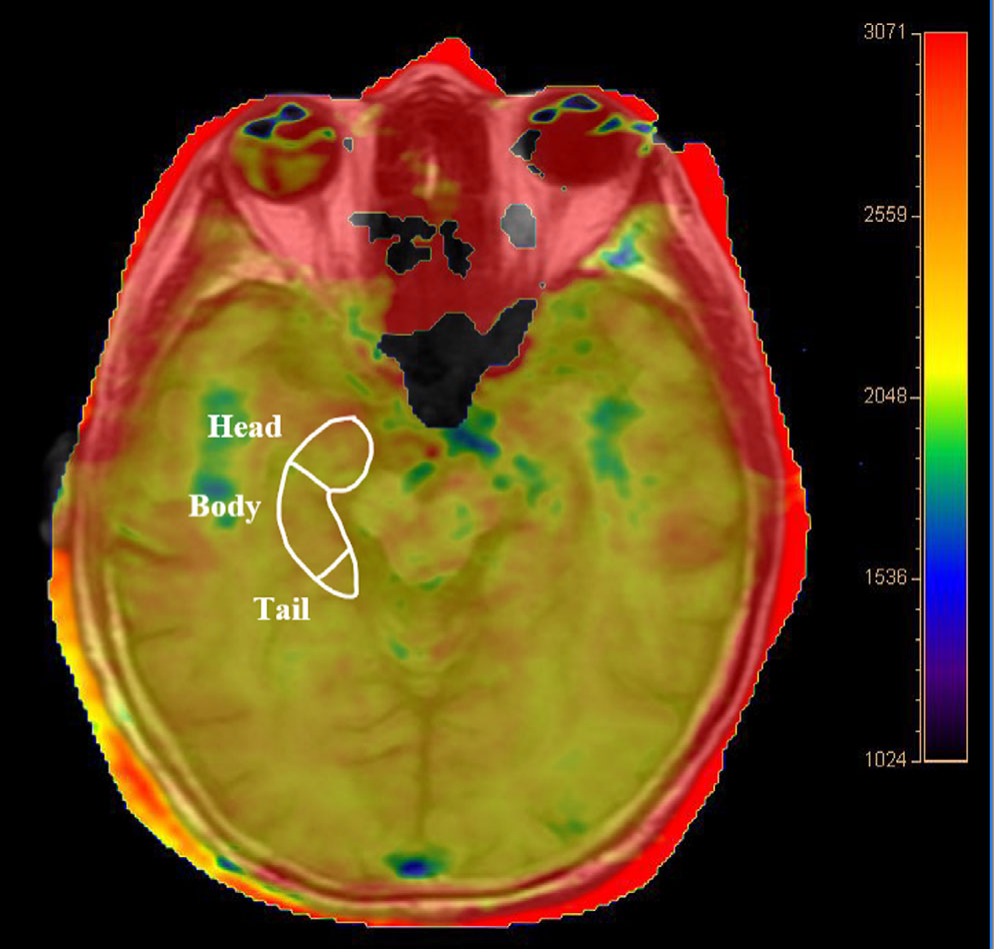
Representative regions of interest (ROIs) for amide proton transfer‐weighted (APTw) analysis. Delineation of the right hippocampal ROI on the T1‐weighted image with a functional map of the hippocampal APTw imaging overlay at the same level (hippocampus head, body and tail are shown).

APTw data were imported into the post‐processing ‘IntelliSpace Portal’ software (Philips Healthcare, Best, Netherlands). Using the oblique axial position parallel to the bilateral hippocampal image, regions of interest (ROI) were manually selected in the head, body and tail of both hippocampi according to the segmentation scheme which followed Daugherty et al.^28^ (Figure 2). The head, body and tail make up approximately 30%, 50% and 20% of the total hippocampus, respectively.^29^ ROIs were uniform measuring 8 mm^2^ in diameter. The APTw signal intensity (SI, %) at each hippocampal subregion (head, body and tail) was measured twice by a senior neuroradiologist with 15 years of experience, with the two measurements separated by a 7‐day interval to minimise the possible impact of the first measurement. APTw signal intensity (SI, %) was measured twice at each site. APTw images consisted of the MTR asymmetry (MTR_asym_) map at a 3.5 ppm offset, defined as: MTR_asym_ (3.5 ppm) = MTR (3.5 ppm) − MTR (−3.5 ppm) = [Ssat (−3.5 ppm) − Ssat (3.5 ppm)]/S0.

### 2.5 Statistical analyses

Statistical analyses were performed using GraphPad Prism (Version 10, La Jolla, USA) and MedCalc (Version 22.023, Mariakerke, Belgium). Continuous variables are presented as mean ± SD for normally distributed data or median and interquartile range (IQR) for non‐normally distributed data. The normality of all continuous variables was assessed using the Kolmogorov–Smirnov test. Categorical data are presented as counts or percentages and were compared using the Chi‐square test. For group comparisons of continuous variables, a one‐way analysis of variance (ANOVA) was applied if the data met the assumptions of normality and homogeneity of variance, followed by Tukey honestly significant difference (HSD) test for post hoc pairwise comparisons. When the normality assumption was violated (as was the case for several key variables, including APTw signal intensity and cognitive scores), the non‐parametric Kruskal–Wallis *H* test was employed for comparisons across the three groups (T2DM‐MCI, T2DM‐nMCI and CG‐nMCI), with Dunn test and Bonferroni correction used for post‐hoc analyses. Analysis of covariance (ANCOVA) was used for specific comparisons: (1) to test for between‐group differences, APTw values from each hippocampal subregion (head, body and tail) were compared between the CG‐nMCI and each patient group (T2DM‐nMCI and T2DM‐MCI); (2) to test for within‐group differences in APTw signal across the hippocampal subregions (head, body and tail) within the T2DM‐nMCI or T2DM‐MCI groups. Differences in signal intensity between the left and right hippocampal subregions were assessed using paired *t* tests or their non‐parametric equivalents, as appropriate. Correlations between APTw values and neuropsychological scores were analysed using Pearson correlation coefficient if both variables were normally distributed (assessed via the Shapiro–Wilk test); otherwise, Spearman rank correlation was applied. The intra‐rater reliability of APTw measurements was evaluated using the Bland–Altman method, with results quantified by systematic bias (mean difference) and random error (95% limits of agreement). To account for baseline cognitive score differences, a multiple linear regression was performed controlling for both baseline FBG and HbA1c. The diagnostic performance of hippocampal APTw signal intensity for identifying MCI was assessed using receiver operating characteristic (ROC) curves, with the area under the curve (AUC) reported. In all analyses, a two‐sided *p*‐value of <0.05 was considered statistically significant. For figures and post hoc tests, significance levels are denoted as follows: *p < 0.05, **p < 0.01, ***p < 0.001 and ****p < 0.0001.

## 3 RESULTS

### 3.1 Demographic, clinical and laboratory variables of the participants

Of the 42 patients and 20 controls initially recruited, 19 T2DM‐MCI patients, 8 T2DM‐nMCI patients and 11 CG‐nMCI participants were ultimately included in the analysis (Figure 1). Baseline demographic characteristics showed no significant intergroup differences (Table 2). The T2DM‐MCI and T2DM‐nMCI groups had comparable clinical histories but significantly differed in FBG (*F* = 5.28, *p* = 0.009) and HbA1c (*t* = 3.14, *p* = 0.004).

**TABLE 2:** The baseline demographic, laboratory and clinical characteristics of type 2 diabetes mellitus (T2DM) and control group without mild cognitive impairment (CG‐nMCI) groups.

Variable mean (SD) unless otherwise shown	T2DM‐MCI	T2DM‐nMCI	CG‐nMCI	Statistics	Effect sizes	*p* Value
Age (year)	60.9 ± 1.0	58.7 ± 2.5	58.45 ± 1.9	0.99^a^	0.038	0.391
Male/female	18/10	5/3	4/7	0.80^a^	0.032	0.455
Educational level (year)	10.18 ± 0.52	12.86 ± 0.88	10.64 ± 0.64	3.05^a^	0.124	0.058
Medical history						
Duration of diabetes (year)	11.04 ± 1.37	10.00 ± 2.40	N/A	0.39^b^	0.144	0.697
Hypertension, *n* (%)	9 (32.1)	4 (50.0)	3 (27.3)	0.57^a^	0.025	0.572
Laboratory investigations						
FBG (mmol/L)	9.04 ± 0.76	10.25 ± 1.52	4.71 ± 0.11	5.28^a^	0.205	0.009^c^
HbA1c (%)	5.93 ± 0.66	10.87 ± 1.36	N/A	3.14^b^	0.230	0.004^c^
TG (mmol/L)	3.87 ± 0.42	2.80 ± 0.68	2.64 ± 0.29	4.63^a^	0.205	0.051
TC (mmol/L)	7.16 ± 0.51	5.22 ± 1.12	5.18 ± 0.24	3.32^a^	0.152	0.053

Abbreviations: FBG, fasting blood‐glucose; HbA1c, glycosylated haemoglobin; N/A, not applicable; SD, standard deviation; T2DM‐MCI, T2DM with MCI; T2DM‐nMCI, T2DM without MCI; TC, total cholesterol; TG, triglyceride.

^a^ Comparisons among T2DM‐MCI group, T2DM‐nMCI group and CG‐nMCI group were made by the *F*‐test (ANOVA), effect sizes: *R* ^2^.

^b^ T2DM‐MCI group was compared to T2DM‐nMCI group using a *t* test, effect sizes: Cohen *d*.

^c^ Indicating *p* < 0.05 (applied Bonferroni correction).

### 3.2 Neuropsychological variables of the participants

Following assessment using the Hamilton Depression Scale, all research participants were confirmed to be free from depression. A multiple linear regression analysis, adjusted for baseline FBG and HbA1c, confirmed that group membership (T2DM‐MCI vs. T2DM‐nMCI) was a strong, independent predictor of MoCA score (*β* = −5.21, 95% confidence interval [CI]: −6.89 to −3.53; *p* < 0.0001). After simultaneous adjustment for both FBG and HbA1c: T2DM‐MCI patients scored lower than T2DM‐nMCI (WMS‐DST‐T: *β* = −2.47, 95% CI: −4.81 to −0.13; *p* = 0.039, WMS‐DST‐B: *β* = −1.62, 95% CI: −2.75 to −0.50; *p* = 0.006, SVF: *β* = −3.71, 95% CI: −6.83 to −0.58; *p* = 0.022). T2DM‐MCI patients scored lower than CG‐nMCI groups (MoCA, AVLT‐H‐D, WMS‐DST‐T and SVF; all *p* ≤ 0.032), while unexpectedly, T2DM‐nMCI patients outperformed CG‐nMCI on WMS‐DST‐T/B (*p* ≤ 0.027) (Table 3, Supporting Information S1: ESM A‐1a–e).

**TABLE 3:** Neuropsychological characteristics of type 2 diabetes mellitus (T2DM) and control group without mild cognitive impairment (CG‐nMCI) groups.

Variable	T2DM‐MCI	T2DM‐nMCI	CG‐nMCI	*F*‐value	Effect sizes (*R* ^2^)	*p* Value
MoCA	21.83 ± 0.37	27.14 ± 0.34	26.27 ± 0.49	39.21	0.635	<0.0001^b^
	<0.0001^a^, ^b^, ^c^	0.215^d^	<0.0001^b^, ^e^			
MMSE	26.80 ± 0.35	27.00 ± 0.53	27.64 ± 0.47	0.87	0.037	0.425
AVLT‐H‐I	15.43 ± 0.74	16.43 ± 1.84	19.00 ± 1.43	2.73	0.108	0.076
AVLT‐H‐D	4.37 ± 0.37	5.86 ± 1.06	6.09 ± 0.55	8.49	0.152	0.0004^b^
	0.170^a^, ^c^	0.828^d^	0.001^b^, ^e^			
WMS‐DST‐T	11.90 ± 0.52	15.14 ± 0.40	12.73 ± 0.51	42.11	0.181	<0.0001^b^
	0.039^a^, ^b^, ^c^	<0.0001^b^, ^d^	0.010^b^, ^e^			
WMS‐DST‐F	8.37 ± 0.22	9.29 ± 0.36	8.55 ± 0.34	1.82	0.075	0.173
WMS‐DST‐B	3.87 ± 0.21	5.86 ± 0.59	4.18 ± 0.40	7.24	0.244	0.002^b^
	0.006^a^, ^b^, ^c^	0.027^b^, ^d^	0.457^e^			
TMT‐A (s)	55.67 ± 2.64	48.71 ± 3.37	51.27 ± 4.22	0.94	0.040	0.398
SVF	16.97 ± 0.66	20.29 ± 1.02	19.73 ± 0.99	4.32	0.161	0.019^b^
	0.022^a^, ^b^, ^c^	0.712^d^	0.032^b^, ^e^			

Abbreviations: AVLT‐H, Auditory Verbal Learning Test‐Huashan version; AVLT‐H‐D, Auditory Verbal Learning Test‐Huashan version‐delayed recall; AVLT‐H‐I, Auditory Verbal Learning Test‐Huashan version‐immediate recall; MoCA, Montreal Cognitive Assessment; MMSE, Mini‐Mental State Examination; T2DM‐MCI group, type 2 diabetes mellitus with MCI; T2DM‐nMCI group, T2DM without MCI; TMT‐A, Trail‐Making Test‐part A; SVF, semantic verbal fluency; WMS‐DST‐B, Wechsler Memory Scale‐Digit Span Test‐Backward; WMS‐DST‐F, Wechsler Memory Scale‐Digit Span Test‐Forward; WMS‐DST‐T, Wechsler Memory Scale‐ Digit Span Test‐total score.

^a^ Indicating *p* value of T2DM‐MCI group compared to T2DM‐nMCI group.

^b^ Indicating *p* < 0.05 (applied Bonferroni correction).

^c^ Association measures are derived from a multiple linear regression model that included group membership, fasting blood‐glucose and glycosylated haemoglobin as independent variables in T2DM‐MCI and T2DM‐nMCI groups.

^d^ Indicating *p* value of T2DM‐nMCI group compared to CG‐nMCI.

^e^ Indicating *p* value of T2DM‐MCI group compared to CG‐nMCI.

### 3.3 Comparison of APTw signal intensity (%)

#### 3.3.1 The hippocampal subregion APTw values for the CG‐nMCI group

The CG‐nMCI group exhibited mean APTw values of 1.38 ± 0.18% (head), 1.11 ± 0.16% (body) and 1.21 ± 0.17% (tail) in the left hippocampus; and 1.60 ± 0.18% (head), 1.37 ± 0.10% (body) and 1.21 ± 0.10% (tail) in the right hippocampus (Table 4).

**TABLE 4:** Amide proton transfer‐weighted signal intensity in the hippocampi of type 2 diabetes mellitus (T2DM) and control group without mild cognitive impairment (CG‐nMCI) groups.

	Left			Right		
	Head	Body	Tail	Head	Body	Tail
T2DM‐MCI	2.50 ± 0.16	1.54 ± 0.12	1.35 ± 0.12	2.01 ± 0.17	1.56 ± 0.12	1.37 ± 0.07
	0.934^a^, ^b^	0.318^a^, ^b^	0.658^a^, ^b^	0.284^a^, ^b^	0.289^a^, ^b^	0.258^a^, ^b^
T2DM‐nMCI	1.83 ± 0.23	1.79 ± 0.20	1.35 ± 0.26	1.64 ± 0.22	1.33 ± 0.11	1.73 ± 0.20
	<0.0001^c^, ^d^	<0.0001^c^, ^d^	0.476^c^	0.890^c^	0.788^c^	<0.0001^c^, ^d^
CG‐nMCI	1.38 ± 0.18	1.11 ± 0.16	1.21 ± 0.17	1.60 ± 0.18	1.37 ± 0.10	1.21 ± 0.10
	0.004^d^, ^e^	< 0.0001^d^, ^e^	0.340^e^	0.177^e^	0.332^e^	0.0004^d^, ^e^
*F*‐statistic	51.78	29.16	0.45	1.37	0.97	31.76
Effect sizes (*R* ^2^)	0.497	0.146	0.011	0.064	0.047	0.160
*p* Value	<0.0001^d^	<0.0001^d^	0.637	0.267	0.388	0.001^d^

Abbreviations: MCI, mild cognitive impairment; T2DM‐MCI group, T2DM with MCI; T2DM‐nMCI group, T2DM without MCI.

^a^ Indicating *p* value of T2DM‐MCI group compared to T2DM‐nMCI group.

^b^ Association measures are derived from a multiple linear regression model that included group membership, fasting blood‐glucose and glycosylated haemoglobin as independent variables in T2DM‐MCI and T2DM‐nMCI groups.

^c^ Indicating *p* value of T2DM‐nMCI group compared to CG‐nMCI.

^d^ Indicating *p* < 0.05 (applied Bonferroni correction).

^e^ Indicating *p* value of T2DM‐MCI group compared to CG‐nMCI.

#### 3.3.2 Comparison of hippocampal subregional APTw values within the T2DM‐MCI and T2DM‐nMCI groups

**FIGURE 3:**
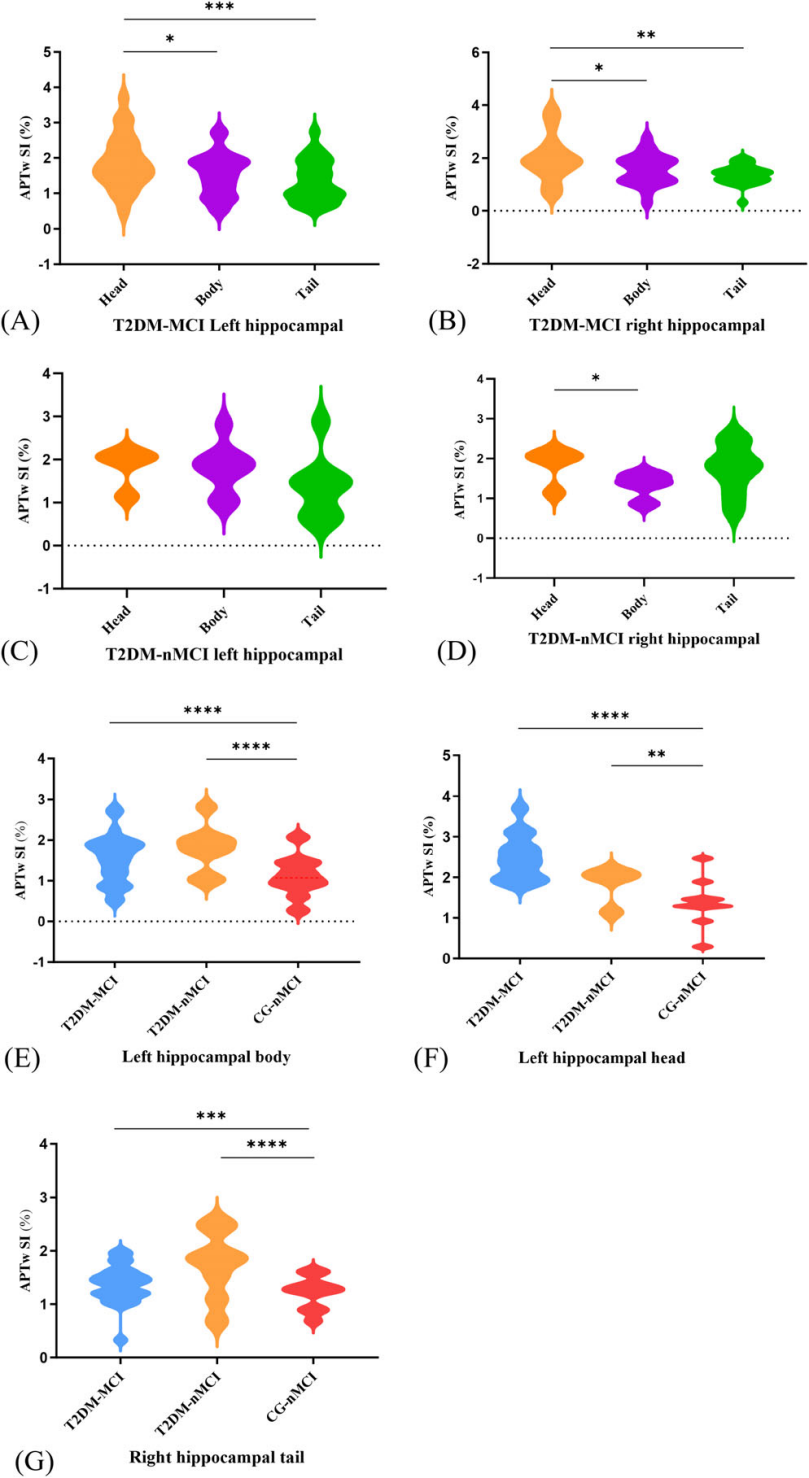
Box‐plot and violin plots show the mean (± standard deviation) of signal intensities (SI) acquired by the amide proton transfer‐weighted (APTw) sequence in the community‐based controls without mild cognitive impairment (CG‐nMCI) and type 2 diabetes mellitus (T2DM) patients with or without MCI. The APTw SI of the head, body and tail of the left hippocampus (A) and the right hippocampus (B) were statistically significant in the T2DM‐MCI group. In the T2DM‐nMCI group, APTw SI showed no significant differences across the left hippocampus (head, body and tail) (C), but significant variations in the right hippocampus (head and body) (D). The SI of the left hippocampal head (E), left hippocampal body (F) and right hippocampal tail (G) were statistically different between three groups (*p < 0.05, **p < 0.01, ***p < 0.001 and ****p < 0.0001).

In T2DM‐MCI patients, APTw signal intensity exhibited a head‐to‐tail gradient in both hippocampi, with heads (left: 2.50 ± 0.16%; right: 2.01 ± 0.17%) showing significantly higher values than bodies (left: 1.54 ± 0.12%, *t* = 2.46, *p* = 0.016, Cohen *d* = 0.51; right: 1.56 ± 0.12%, *t* = 2.09, *p* = 0.042, Cohen *d* = 0.62) and tails (left: 1.35 ± 0.12%, *t* = 3.83, *p* = 0.0002, Cohen *d* = 0.79; right: 1.37 ± 0.07%, *t* = 3.27, *p* = 0.002, Cohen *d* = 0.96), but no body‐tail differences (*p* > 0.130) (Figure 3A,B). In T2DM‐nMCI patients, only the right hippocampal head showed higher signals than the body (*t* = 2.69, *p* = 0.018, Cohen *d* = 1.44), with no other significant regional differences (all *p* > 0.123) in either hemisphere (Figure 3C,D).

#### 3.3.3 Comparison of hippocampal subregional APTw values among T2DM‐MCI, T2DM‐nMCI and CG‐nMCI groups

T2DM‐MCI patients showed significantly higher APTw signal intensity in the left hippocampal head (2.50 ± 0.16%) compared to CG‐nMCI (1.38 ± 0.18%, *t* = 9.80, *p* < 0.0001, Cohen *d* = 2.07). In the left body, T2DM‐MCI had higher signals than CG‐nMCI (1.11 ± 0.16%, *t* = 5.91, *p* < 0.0001, Cohen *d* = 0.71). Compared to the CG‐nMCI group, T2DM‐nMCI patients exhibited significantly higher APTw signal intensity in both the left hippocampal head (1.83 ± 0.23% vs. 1.38 ± 0.18%; *t* = 3.02, Cohen *d* = 0.82, *p* = 0.004) and body (1.79 ± 0.20% vs. 1.11 ± 0.16%; *t* = 8.02, Cohen *d* = 1.35, *p* < 0.001). Similar patterns occurred in the right tail (T2DM‐MCI: 1.37 ± 0.07% vs. CG‐nMCI: 1.21 ± 0.10%, *t* = 3.57, *p* = 0.0004, Cohen *d* = 0.05). The T2DM‐nMCI group consistently showed higher signals than CG‐nMCI across right hippocampal tail (*t* = 6.90, *p* < 0.0001, Cohen *d* = 1.16). After simultaneous adjustment for baseline FBG and HbA1c, the analysis revealed no statistically significant differences in APTw signal intensity in any hippocampal subregions between the T2DM‐MCI and T2DM‐nMCI groups (all *p* > 0.25). (Table 4) (Supporting Information S1: ESM A‐1f–k, Figure 3E–G).

#### 3.3.4 Within‐group analysis of bilateral hippocampal APTw values in T2DM‐MCI and T2DM‐nMCI groups

Significant left–right asymmetry was observed only in the hippocampal heads of T2DM‐MCI patients (*t* = 3.04, *p* = 0.003, Cohen *d* = 0.70), with no significant differences in T2DM‐nMCI (*t* = 0.71, *p* = 0.488, Cohen *d* = 0.38) and CG‐nMCI (*t* = 0.89, *p* = 0.388, Cohen *d* = 0.42). No interhemispheric differences were found in hippocampal bodies (T2DM‐MCI: *t* = 0.09, *p* = 0.927, Cohen *d* = 0.03; T2DM‐nMCI: *t* = 1.99, *p* = 0.066, Cohen *d* = 1.07; CG‐nMCI: *t* = 1.41, *p* = 0.177, Cohen *d* = 0.66) or tails (T2DM‐MCI: *t* = 0.16, *p* = 0.874, Cohen *d* = 0.05; T2DM‐nMCI: *t* = 1.11, *p* = 0.288, Cohen *d* = 0.59; CG‐nMCI: *t* = 0.04, *p* = 0.967, Cohen *d* = 0.02).

### 3.4 Correlation of neuropsychological variables and APTw signal intensity in the hippocampi of T2DM‐MCI and T2DM‐nMCI patients

**FIGURE 4:**
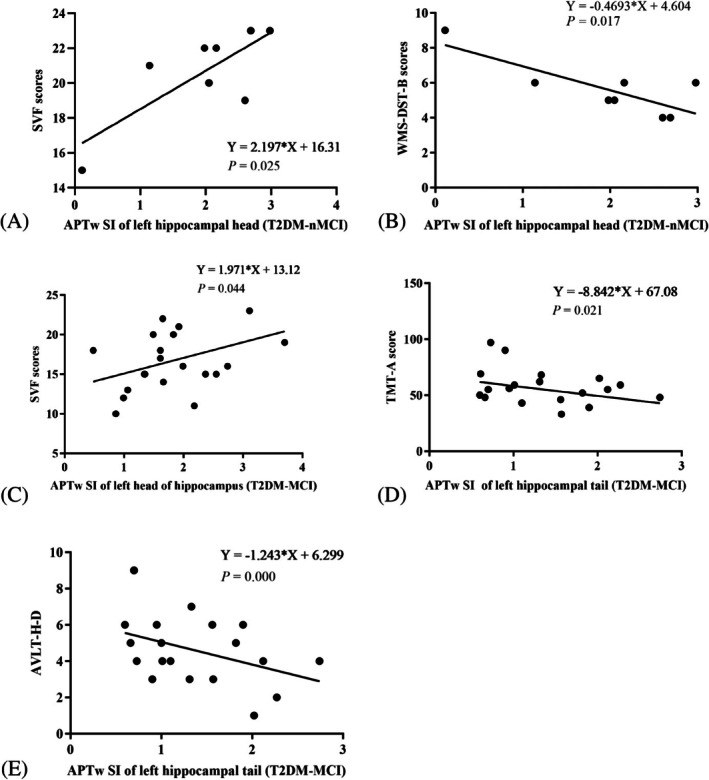
Correlation analysis of amide proton transfer‐weighted (APTw) signal intensities (SI) in left hippocampus and neuropsychological variables of type 2 diabetes mellitus without mild cognitive impairment (T2DM‐nMCI) and T2DM with mild cognitive impairment (T2DM‐MCI) group. Data from the T2DM‐nMCI group (*n* = 8) were analysed using Pearson correlation. The results revealed a strong positive correlation between semantic verbal fluency (SVF) score and APTw SI (%) in the left hippocampal head (*r* = 0.770, *p* = 0.025, [A]). Additionally, the Wechsler Intelligence Scale‐digit Span Test‐Backward (WMS‐DST‐B) score was negatively correlated with APTw SI (%) in the left hippocampal head (*r* = −0.802, *p* = 0.017, [B]). *p* < 0.05 was considered statistically significant. Data from the T2DM‐MCI group (*n* = 19) were analysed using Pearson correlation. The results revealed a mild positive correlation between SVF score and APTw SI (%) in the left hippocampal head (*r* = 0.414, *p* = 0.044, [C]). There is a mild negative correlation between Trail‐Making Test‐A (TMT‐A) score (*r* = −0.333, *p* = 0.021, [D])/Auditory Verbal Learning Test‐Huashan version‐delayed recall (AVLT‐H‐D) (*r* = −0.376, *p <* 0.001, [E]) and APTw SI (%) in the left hippocampal tail. *p* < 0.05 was considered statistically significant.

In the T2DM‐nMCI group, SVF scores were positively correlated with the APTw SI of the left hippocampal head (*r* = 0.770, *p* = 0.025), WMS‐DST‐B scores were negatively correlated with APTw SI in the left hippocampal head (*r* = −0.802, *p* = 0.017) (Supporting Information S1: ESM A‐2). In the T2DM‐MCI group, SVF scores were positively correlated with the APTw SI of the left hippocampal head (*r* = 0.414, *p* = 0.044). TMT‐A scores were negatively correlated with the APTw SI of the left hippocampal tail (*r* = −0.333, *p* = 0.021), and AVLT‐H‐D scores were negatively correlated with the APTw signal intensity of the left hippocampal tail (*r* = −0.376, *p* = 0.000) (Supporting Information S1: ESM A‐3, Figure 4A–E).

### 3.5 Potential diagnostic value of hippocampal APTw in identifying mild cognitive impairment among T2DM‐nMCI

**FIGURE 5:**
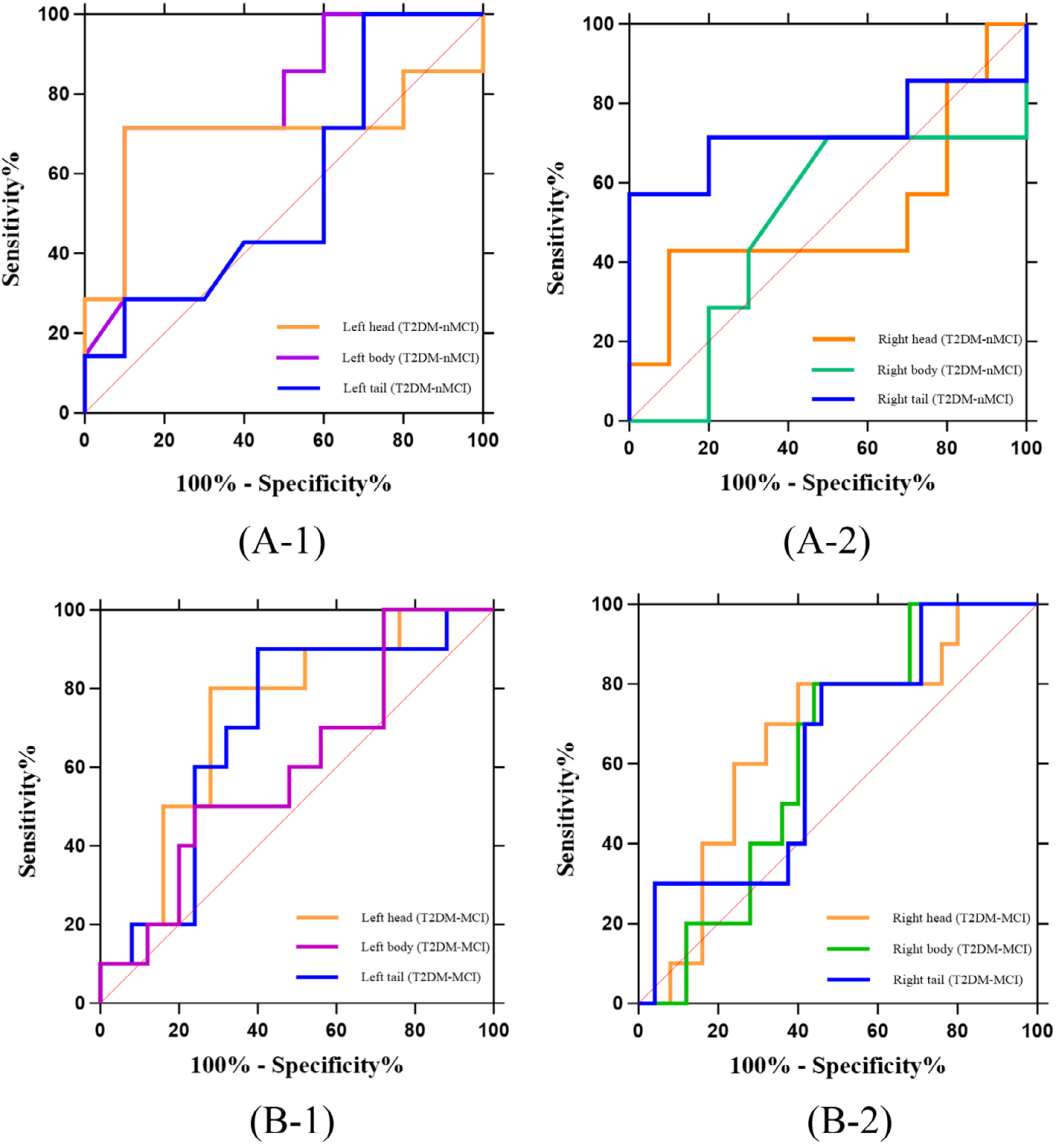
Diagnostic performance of amide proton transfer‐weighted (APTw) signal intensities (SI) in bilateral hippocampal head, body and tail of type 2 diabetes mellitus without mild cognitive impairment (T2DM‐nMCI) and T2DM with mild cognitive impairment (T2DM‐MCI). (A1‐2) The receiver operating characteristic (ROC) curve analysis of the T2DM‐nMCI group revealed that the APTw SI (%) in the left body of hippocampus demonstrated statistically significant diagnostic efficacy (area under the curve [AUC] = 0.793, *p* = 0.045), whereas the APTw SI (%) in other hippocampal regions showed non‐significant diagnostic value (AUCs = 0.700, 0.564, 0.514, 0.500 and 0.729; all *p* > 0.05). (B1‐2) The ROC curve analysis of the T2DM‐MCI group revealed that the APTw SI (%) in the left head of hippocampus demonstrated statistically significant diagnostic efficacy (AUC = 0.732, *p* = 0.034), whereas the APTw SI (%) in other hippocampal regions showed non‐significant diagnostic value (AUCs = 0.696, 0.604, 0.668, 0.624 and 0.638; all *p* > 0.05).

The left hippocampal body showed potential diagnostic value for T2DM without MCI (AUC = 0.793, 95% CI: 0.5673–1.000, *p* = 0.045). In contrast, several other AUC measured in this study showed limited diagnostic utility: the left hippocampal head (AUC = 0.700), left hippocampal tail (AUC = 0.564), right hippocampal head (AUC = 0.514), right hippocampal body (AUC = 0.500) and right hippocampal tail (AUC = 0.729) all exhibited non‐significant discriminative power (*p* > 0.05) (Table 5, Figure 5A1‐2).

**TABLE 5:** Diagnostic value for type 2 diabetes mellitus without mild cognitive impairment in different regions of hippocampus.

Variable	L‐head	L‐body	L‐tail	R‐head	R‐body	R‐tail
AUC	0.700	0.793	0.564	0.514	0.500	0.729
95% CI	0.3963–1.004	0.5673–1.000	0.2776–0.851	0.2006–0.8279	0.1955–0.8045	0.4358–1.021
*p*	0.172	0.045^a^	0.661	0.922	0.999	0.118

Abbreviations: AUC, area under the curve; CI, confidence interval; L, left; R, right.

^a^ Indicating *p* < 0.05.

### 3.6 Potential diagnostic value of hippocampal APTw in identifying mild cognitive impairment risk in T2DM‐MCI

The AUC of ROC curves for the left hippocampal head demonstrated higher diagnostic value for MCI in T2DM, with an AUC of 0.732 (95% CI: 0.5546–0.9094, *p* = 0.034). However, other AUCs, including the left hippocampal body (AUC = 0.696), left hippocampal tail (AUC = 0.604), right hippocampal head (AUC = 0.668), right hippocampal body (AUC = 0.624) and right hippocampal tail (AUC = 0.638), showed unsatisfactory performance (*p* > 0.05) (Table 6, Figure 5B1‐2).

**TABLE 6:** Diagnostic value for type 2 diabetes mellitus with mild cognitive impairment in different regions of hippocampus.

Variable	L‐head	L‐body	L‐tail	R‐head	R‐body	R‐tail
AUC	0.732	0.696	0.604	0.668	0.624	0.638
95% CI	0.5546–0.9094	0.5068–0.8852	0.3997–0.8083	0.4725–0.8635	0.4382–0.8098	0.4393–0.8357
*p*	0.034^a^	0.074	0.342	0.125	0.258	0.212

Abbreviations: AUC, area under the curve; CI, confidence interval; L, left; R, right.

^a^ Indicating *p* < 0.05.

### 3.7 Diagnostic performance of hippocampal APTw signal intensity in distinguishing T2DM‐MCI from T2DM‐nMCI

The AUC values for various hippocampal subregions—including the left hippocampal head (AUC = 0.531), left hippocampal body (AUC = 0.651), left hippocampal tail (AUC = 0.623), right hippocampal head (AUC = 0.617), right hippocampal body (AUC = 0.680) and right hippocampal tail (AUC = 0.637)—demonstrated unsatisfactory performance in discriminating between T2DM‐MCI and T2DM‐nMCI groups (all *p* > 0.05). Among these, only the right hippocampal body exhibited a trend towards significance (AUC = 0.680, 95% CI: 0.4902–0.8698, *p* = 0.151) (Supporting Information S1: ESM A‐4).

### 3.8 Comparison of Bland–Altman consistency of the left hippocampal head in three groups

The mean difference of APTw values in the Bland–Altman analysis of −0.11 (95% CI = 0.61 to −0.84) showed a strong agreement between the two measurements (Supporting Information S1: ESM B).

## 4 DISCUSSION

This exploratory study reveals a spatially evolving pattern of hippocampal APTw alterations in T2DM, suggesting distinct subregional involvement during cognitive decline. Elevated signals in the left hippocampal head and right hippocampal tail were linked to specific cognitive deficits, while the differential diagnostic potential of the left hippocampal head (for T2DM‐MCI) and body (for T2DM‐nMCI) underscores APTw's promise in tracking disease progression.

After simultaneous adjustment for both FBG and HbA1c, this study found that the T2DM‐MCI group scored significantly lower than the other two groups on the total scores of the MoCA and WMS‐DST. WMS‐DST is a test primarily used to assess working memory. MCI patients can generally maintain their daily functioning, but they often experience impairments in memory, attention, language, visuospatial skills and calculation. Among these, memory is typically the first function to deteriorate, making it significant for early diagnosis of MCI.^30^ Additionally, compared to the CG‐nMCI, the T2DM‐nMCI group scored lower on the AVLT‐H‐D, but higher on the WMS‐DST‐T and WMS‐DST‐B. Our result indicated that verbal memory might plausibly be influenced first before the onset of MCI in T2DM patients. Typically, for preclinical AD, verbal memory is affected initially.^31^ Studies have proven that AD and DACD share the same cognitive impairment process.^32^, ^33^ More studies are needed in the future to confirm or refute this.

In the T2DM‐MCI group, APTw values showed a stepwise reduction from head‐to‐tail of the bilateral hippocampus, suggesting region‐specific protein accumulation patterns in cognitive impairment progression. In both the T2DM‐nMCI and CG‐nMCI groups, APTw values in the hippocampal heads were higher than those in the body and tail. Elevated APTw values were also observed in AD patients with amnestic MCI (aMCI), consistent with previous APTw imaging studies.^23^, ^34^ The APTw sequence is sensitive to mobile proteins, which have a specific amide proton resonance at a frequency of 3.5 ppm downfield of water; thus, a higher concentration of amide protons theoretically results in higher APTw values. Previous research has shown that T2DM and AD share overlapping pathophysiological mechanisms, such as abnormal protein deposition.^33^ The accumulation of misfolded proteins is a hallmark of neurodegenerative diseases,^35^, ^36^ particularly in the hippocampal region—a structure within the limbic system involved in complex memory functions.^37^ The hippocampus, which is divided into the head, body and tail, plays different roles in different memory functions.^38^ Research has shown that Tau proteins are deposited in the hippocampal subregions in a specific neuroanatomical order.^39^ Our results suggested an increased concentration of mobile proteins in the hippocampal head compared to other areas, consistent with previous studies. Furthermore, we found that the T2DM‐nMCI group had elevated APTw in the left hippocampal body and right hippocampal tail compared to the CG‐nMCI, suggesting that these hippocampal regions may exhibit measurable alterations prior to the onset of overt cognitive symptoms. The hippocampal head is primarily associated with short‐term memory and learning, and memory function is often the first to be impaired,^40^, ^41^ our study is consistent with these findings. In conclusion, our study suggests that APTw changes in different hippocampal regions may signal the neuropathological processes preceding MCI in T2DM. However, as noted in the study limitations, the direct utility of the left hippocampal body as an early diagnostic marker requires further validation through direct comparison between T2DM patients with and without MCI in future longitudinal studies. Nonetheless, these findings highlight the potential role of APTw imaging in identifying early brain changes in T2DM.

Our results suggested that WMS‐DST‐B scores were negatively correlated with the APTw value of the left hippocampal head in T2DM‐nMCI patients. WMS‐DST‐B scores presented working memory. Working memory is a crucial function of the brain for processing and storing information, with the hippocampus playing a vital role in this process.^42^ In the early stages of AD, neuronal damage and the accumulation of abnormal proteins in the hippocampal region can lead to a significant decline in working memory. This decline not only affects the patient's daily living abilities but may also serve as an early indicator of disease progression.^43^ SVF scores showed a positive correlation with APTw values in the left hippocampal head of the T2DM‐nMCI and T2DM‐MCI group, implying that language function and executive function are enhanced—either in a compensatory way or even that the increased intracellular proteins in this region may have a protective or promoting effect.^44^ Our results also showed that TMT‐A and AVLT‐H scores were negatively correlated with the APTw value of the left hippocampal head and tail, respectively, suggesting that intracellular free proteins may diminish attention subsequently and verbal working memory was further aggravated in T2DM‐MCI patients. In AD, the initial stages are marked by episodic memory impairment, which is closely linked to changes in the medial temporal lobe (especially the hippocampal structure), a region crucial for memory processing. As the disease progresses, other cognitive domains, including attention, become affected, reflecting the spread of neuropathological changes to other brain regions.^45^ These results indicate that APTw SI can indirectly reflect the process of cognitive impairment, which may provide a non‐invasive, early imaging biomarker for clinical assessment of cognitive impairment. However, further experimental analysis is needed to identify the specific mobile protein species involved.

ROC analysis revealed potential early signal for T2DM‐MCI: the left hippocampal body (AUC = 0.793) showed some discriminatory power for T2DM‐nMCI patients, while the left hippocampal head (AUC = 0.732) demonstrated higher efficacy in identifying MCI among T2DM. Therefore, we consider APTw SI, particularly in the left hippocampal head and body (supported by AUCs of 0.732 and 0.793, respectively), shows promise as an imaging biomarker for early‐stage DACD in T2DM. The present study highlights the distinct value of APTw imaging compared to standard cognitive assessments. Neuropsychological tests are indispensable for quantifying cognitive status, but our findings suggest that APTw imaging provides a potential alternative: an objective, quantifiable biomarker of the underlying protein‐level pathology in specific brain subregions. This capability to probe the disease's mechanistic basis, rather than just its symptomatic output, positions APTw as a promising tool for the early detection and pathological stratification of DACD, potentially enabling interventions at a stage when they might be most effective. However, given the cross‐sectional nature of this study, the clinical utility of these markers for predicting future MCI onset requires validation. To this end, our group is planning a prospective longitudinal follow‐up study to directly assess the predictive performance of these biomarkers for MCI. In our analysis, the hippocampal subregions generally showed unsatisfactory discriminatory power between T2DM‐MCI and T2DM‐nMCI groups. Among them, only the right hippocampal body demonstrated a potential trend, though not statistically significant (*p* = 0.151). This could be attributed to our limited sample size. We will increase the sample size in future studies to further verify these findings.

We found that after simultaneous adjustment for both FBG and HbA1c, no statistically significant differences in APTw signals were observed in any subregions of the hippocampus between the T2DM‐MCI and T2DM‐nMCI groups (all *p* > 0.25). However, prior to adjustment, differences in APTw signals were detected in the left hippocampal head, left hippocampal body and right hippocampal tail (all *p* < 0.05). As the amide proton transfer (APT) signal is intrinsically influenced by complex factors including tissue pH, intracellular protein concentration and metabolic state, it may also be affected by blood glucose levels. Our limited sample size likely amplified the impact of these confounding factors, reducing statistical power and potentially masking genuine effects. Therefore, the initial unadjusted differences may reflect a combination of pathology and confounding metabolic changes. A future study with an expanded cohort will help clarify these observations.

Collectively, our findings extend the existing literature in three key areas. Firstly, we have successfully translated APTw imaging, a technique previously applied in AD,^23^, ^34^, ^36^ to a T2DM sample, confirming its sensitivity to protein‐level changes in this population. Secondly, the subregion‐specific hippocampal alterations we observed—particularly in the left hippocampal body of T2DM‐nMCI patients—provide novel, spatially resolved in vivo evidence supporting the shared proteinopathy hypothesis between T2DM and AD,^33^ while suggesting a potentially distinct early spatial pattern. Most critically, the significant attenuation of APTw differences after adjusting for glycaemic markers highlights the essential confounding role of metabolic status. This highlights that in T2DM, advanced imaging biomarkers like APTw must be interpreted within the integrated framework of metabolic‐neurodegenerative interplay, a crucial consideration for future research and clinical translation.

This study had several limitations. Firstly, the sample size was small and geographically restricted, as all participants were recruited from a single hospital, which may limit the generalisability of the results. Secondly, our study regarding alterations in protein and peptide concentrations remains hypothetical; further laboratory‐based studies—such as animal models or in vitro experiments—are required to confirm the exact types and quantities of proteins involved. Thirdly, MCI was not differentiated into aMCI and non‐amnestic MCI (non‐aMCI) subtypes. Furthermore, the absence of long‐term diagnostic verification of MCI status represents a notable constraint, as the early stage of cognitive impairment necessitates repeating assessment to ensure cohort stability. To address these constraints, future research should recruit a larger and more diverse cohort across multiple centres to enhance the external validity of the findings. Furthermore, longitudinal studies are warranted to monitor the progression of cognitive function and its biochemical correlates over an extended period, which would provide valuable insights into the dynamics of MCI in T2DM patients.

## 5 CONCLUSION

Our study suggested that T2DM‐nMCI and T2DM‐MCI patients had higher APTw values in specific hippocampal subregions. Specifically, the APTw signal in the left hippocampal body demonstrated potential for identifying T2DM patients at risk of future cognitive decline, whereas the APTw signal in the left hippocampal head was a more direct indicator of existing MCI in T2DM patients. These findings suggest that APTw values may serve as early, non‐invasive biomarkers for DACD, potentially enabling earlier clinical intervention.

## CONFLICT OF INTEREST STATEMENT

The authors declare that they have no known competing financial interests or personal relationships that could have appeared to influence the work reported in this paper.

## Supporting information


**Data S1.** Supporting Information.

## Data Availability

The data that support the findings of this study are available from the corresponding author upon reasonable request.

## References

[1] Chatterjee S , Khunti K , Davies MJ . Type 2 diabetes. Lancet. 2017;389(10085):2239‐2251. doi:10.1016/S0140-6736(17)30058-2 28190580

[2] Magliano DJ , Boyko EJ , IDF Diabetes Atlas 10th edition Scientific Committee . IDF Diabetes Atlas. 10th ed. International Diabetes Federation; 2021.

[3] Atlantis E , Fahey P , Foster J . Collaborative care for comorbid depression and diabetes: a systematic review and meta‐analysis. BMJ Open. 2014;4(4):e004706. doi:10.1136/bmjopen-2013-004706 PMC3987739 24727428

[4] Dao L , Choi S , Freeby M . Type 2 diabetes mellitus and cognitive function: understanding the connections. Curr Opin Endocrinol Diabetes Obes. 2023;30(1):7‐13. doi:10.1097/MED.0000000000000783 36385094

[5] Koekkoek PS , Kappelle LJ , van den Berg E , Rutten GE , Biessels GJ . Cognitive function in patients with diabetes mellitus: guidance for daily care. Lancet Neurol. 2015;14(3):329‐340. doi:10.1016/S1474-4422(14)70249-2 25728442

[6] You Y , Liu Z , Chen Y , et al. The prevalence of mild cognitive impairment in type 2 diabetes mellitus patients: a systematic review and meta‐analysis. Acta Diabetol. 2021;58(6):671‐685. doi:10.1007/s00592-020-01648-9 33417039

[7] Petersen RC , Lopez O , Armstrong MJ , et al. Practice guideline update summary: mild cognitive impairment [RETIRED]: report of the guideline development, dissemination, and implementation subcommittee of the American Academy of Neurology. Neurology. 2018;90(3):126‐135. doi:10.1212/WNL.0000000000004826 29282327 PMC5772157

[8] Whitmer RA . Type 2 diabetes and risk of cognitive impairment and dementia. Curr Neurol Neurosci Rep. 2007;7(5):373‐380. doi:10.1007/s11910-007-0058-7 17764626

[9] Fawns‐Ritchie C , Price J , Deary IJ . Association of functional health literacy and cognitive ability with self‐reported diabetes in the English longitudinal study of ageing: a prospective cohort study. BMJ Open. 2022;12(6):e058496. doi:10.1136/bmjopen-2021-058496 PMC9171267 36691240

[10] Bharadwaj P , Wijesekara N , Liyanapathirana M , et al. The link between type 2 diabetes and neurodegeneration: roles for amyloid‐β, amylin, and tau proteins. J Alzheimer's Dis. 2017;59(2):421‐432. doi:10.3233/JAD-161192 28269785

[11] Wang Y , Gao Y , Wang Y , et al. ApoE4 upregulates GSK‐3β to aggravate Alzheimer‐like pathologies and cognitive impairment in type 2 diabetic mice. CNS Neurosci Ther. 2025;31(9):e70575. doi:10.1111/cns.70575 40905109 PMC12409304

[12] Fang K , Jiao B , Shen L , Luo S . Regulation of tau protein by circCwc27: shared pathogenic mechanisms in type 2 diabetes mellitus and Alzheimer's disease. Brain Inform. 2025;12(1):26. doi:10.1186/s40708-025-00277-8 41037159 PMC12491135

[13] Huang X , Huang S , Fu F , Song J , Zhang Y , Yue F . Characterization of preclinical Alzheimer's disease model: spontaneous type 2 diabetic cynomolgus monkeys with systemic pro‐inflammation, positive biomarkers and developing AD‐like pathology. Alzheimer's Res Ther. 2024;16(1):52. doi:10.1186/s13195-024-01416-9 38459540 PMC10921774

[14] Rowles JE , Keane KN , Gomes Heck T , Cruzat V , Verdile G , Newsholme P . Are heat shock proteins an important link between type 2 diabetes and Alzheimer disease? Int J Mol Sci. 2020;21(21):8204. doi:10.3390/ijms21218204 33147803 PMC7662599

[15] Wijesekara N , Ahrens R , Sabale M , et al. Amyloid‐β and islet amyloid pathologies link Alzheimer's disease and type 2 diabetes in a transgenic model. FASEB J. 2017;31(12):5409‐5418. doi:10.1096/fj.201700431R 28808140

[16] Xie J , Wei Q , Deng H , Li G , Ma L , Zeng H . Negative regulation of Grb10 interacting GYF protein 2 on insulin‐like growth factor‐1 receptor signaling pathway caused diabetic mice cognitive impairment. PLoS One. 2014;9(9):e108559. doi:10.1371/journal.pone.0108559 25268761 PMC4182477

[17] Tan H , Wu Z , Wang H , et al. Refined phosphopeptide enrichment by phosphate additive and the analysis of human brain phosphoproteome. Proteomics. 2015;15(2–3):500‐507. doi:10.1002/pmic.201400171 25307156 PMC4598062

[18] Hussain S , Mubeen I , Ullah N , et al. Modern diagnostic imaging technique applications and risk factors in the medical field: a review. Biomed Res Int. 2022;2022:5164970. doi:10.1155/2022/5164970 35707373 PMC9192206

[19] Zhou J , Payen JF , Wilson DA , Traystman RJ , van Zijl P . Using the amide proton signals of intracellular proteins and peptides to detect pH effects in MRI. Nat Med. 2003;9(8):1085‐1090. doi:10.1038/nm907 12872167

[20] Li S , Chan P , Li C , et al. Changes of amide proton transfer imaging in multiple system atrophy parkinsonism type. Front Aging Neurosci. 2020;12:572421. doi:10.3389/fnagi.2020.572421 33192464 PMC7556302

[21] Zhou J , Blakeley JO , Hua J , et al. Practical data acquisition method for human brain tumor amide proton transfer (APT) imaging. Magn Reson Med. 2008;60(4):842‐849. doi:10.1002/mrm.21712 18816868 PMC2579754

[22] Kogan F , Hariharan H , Reddy R . Chemical exchange saturation transfer (CEST) imaging: description of technique and potential clinical applications. Curr Radiol Rep. 2013;1(2):102‐114. doi:10.1007/s40134-013-0010-3 23730540 PMC3665411

[23] Chen X , Gong T , Chen T , et al. Altered glutamate‐glutamine and amide proton transfer‐weighted values in the hippocampus of patients with amnestic mild cognitive impairment: a novel combined imaging diagnostic marker. Front Neurosci. 2023;17:1089300. doi:10.3389/fnins.2023.1089300 36908797 PMC9995585

[24] Alberti KG , Zimmet PZ . Definition, diagnosis and classification of diabetes mellitus and its complications. Part 1: diagnosis and classification of diabetes mellitus provisional report of a WHO consultation. Diabet Med. 1998;15(7):539‐553. doi:10.1002/(SICI)1096-9136(199807)15:7<539::AID-DIA668>3.0.CO;2-S 9686693

[25] Wahlund LO , Barkhof F , Fazekas F , et al. A new rating scale for age‐related white matter changes applicable to MRI and CT. Stroke. 2001;32(6):1318‐1322. doi:10.1161/01.str.32.6.1318 11387493

[26] Albert MS , DeKosky ST , Dickson D , et al. The diagnosis of mild cognitive impairment due to Alzheimer's disease: recommendations from the National Institute on Aging‐Alzheimer's Association workgroups on diagnostic guidelines for Alzheimer's disease. Alzheimers Dement. 2011;7(3):270‐279. doi:10.1016/j.jalz.2011.03.008 21514249 PMC3312027

[27] Petersen RC , Stevens JC , Ganguli M , Tangalos EG , Cummings JL , DeKosky S . Practice parameter: early detection of dementia: mild cognitive impairment (an evidence‐based review) [RETIRED]. Report of the quality standards subcommittee of the American Academy of neurology. Neurology. 2001;56(9):1133‐1142. doi:10.1212/wnl.56.9.1133 11342677

[28] Daugherty AM , Yu Q , Flinn R , Ofen N . A reliable and valid method for manual demarcation of hippocampal head, body, and tail. Int J Dev Neurosci. 2015;41:115‐122. doi:10.1016/j.ijdevneu.2015.02.001 25660945

[29] Chen KH , Chuah LY , Sim SK , Chee MW . Hippocampal region‐specific contributions to memory performance in normal elderly. Brain Cogn. 2010;72(3):400‐407. doi:10.1016/j.bandc.2009.11.007 20044193

[30] Perry RJ , Watson P , Hodges JR . The nature and staging of attention dysfunction in early (minimal and mild) Alzheimer's disease: relationship to episodic and semantic memory impairment. Neuropsychologia. 2000;38(3):252‐271. doi:10.1016/s0028-3932(99)00079-2 10678692

[31] Lange KL , Bondi MW , Salmon DP , et al. Decline in verbal memory during preclinical Alzheimer's disease: examination of the effect of APOE genotype. J Int Neuropsychol Soc. 2002;8(7):943‐955. doi:10.1017/s1355617702870096 12405546 PMC1621042

[32] Ho G , Takamatsu Y , Wada R , et al. Connecting Alzheimer's disease with diabetes mellitus through amyloidogenic evolvability. Front Aging Neurosci. 2020;12:576192. doi:10.3389/fnagi.2020.576192 33192467 PMC7655535

[33] de Matos AM , de Macedo MP , Rauter AP . Bridging type 2 diabetes and Alzheimer's disease: assembling the puzzle pieces in the quest for the molecules with therapeutic and preventive potential. Med Res Rev. 2018;38(1):261‐324. doi:10.1002/med.21440 28422298

[34] Zhang Z , Zhang C , Yao J , et al. Protein‐based amide proton transfer‐weighted MR imaging of amnestic mild cognitive impairment. Neuroimage Clin. 2020;25:102153. doi:10.1016/j.nicl.2019.102153 31901792 PMC6948365

[35] Soto C , Pritzkow S . Protein misfolding, aggregation, and conformational strains in neurodegenerative diseases. Nat Neurosci. 2018;21(10):1332‐1340. doi:10.1038/s41593-018-0235-9 30250260 PMC6432913

[36] Wyss‐Coray T . Ageing, neurodegeneration and brain rejuvenation. Nature. 2016;539(7628):180‐186. doi:10.1038/nature20411 27830812 PMC5172605

[37] Witter MP , Moser EI . Spatial representation and the architecture of the entorhinal cortex. Trends Neurosci. 2006;29(12):671‐678. doi:10.1016/j.tins.2006.10.003 17069897

[38] Aggleton JP . Multiple anatomical systems embedded within the primate medial temporal lobe: implications for hippocampal function. Neurosci Biobehav Rev. 2012;36(7):1579‐1596. doi:10.1016/j.neubiorev.2011.09.005 21964564

[39] Lace G , Savva GM , Forster G , et al. Hippocampal tau pathology is related to neuroanatomical connections: an ageing population‐based study. Brain. 2009;132(Pt 5):1324‐1334. doi:10.1093/brain/awp059 19321462

[40] Qu H , Ge H , Wang L , Wang W , Hu C . Volume changes of hippocampal and amygdala subfields in patients with mild cognitive impairment and Alzheimer's disease. Acta Neurol Belg. 2023;123(4):1381‐1393. doi:10.1007/s13760-023-02235-9 37043115

[41] Guo QH , Cao XY , Zhou Y , Zhao QH , Ding D , Hong Z . Application study of quick cognitive screening test in identifying mild cognitive impairment. Neurosci Bull. 2010;26(1):47‐54. doi:10.1007/s12264-010-0816-4 20101272 PMC5560380

[42] Karceski S . How a buildup of abnormal proteins in the brain may be the key to understanding Alzheimer disease. Neurology. 2020;95(21):e2951‐e2953. doi:10.1212/WNL.0000000000011044 33229499

[43] Serrano N , López‐Sanz D , Bruña R , et al. Spatiotemporal oscillatory patterns during working memory maintenance in mild cognitive impairment and subjective cognitive decline. Int J Neural Syst. 2020;30(1):1950019. doi:10.1142/S0129065719500199 31522594

[44] Sundstrom JM , Hernández C , Weber SR , et al. Proteomic analysis of early diabetic retinopathy reveals mediators of neurodegenerative brain diseases. Invest Ophthalmol Vis Sci. 2018;59(6):2264‐2274. doi:10.1167/iovs.17-23678 29847632 PMC5935294

[45] Pirogovsky‐Turk E , Filoteo JV , Litvan I , Harrington DL . Structural MRI correlates of episodic memory processes in Parkinson's disease without mild cognitive impairment. J Parkinsons Dis. 2015;5(4):971‐981. doi:10.3233/JPD-150652 26577652 PMC4754077

