# Supplementary material for: Early detection of type 2 diabetes mellitus‐associated cognitive dysfunction: The potential of amide proton transfer‐weighted imaging
Source: Diabetes Obes Metab. 2026 Feb 2;28(4):2859–70. doi: 10.1111/dom.70468 (PMC12992161; doi:10.1111/dom.70468)
Supplement: Supplementary file 1 — Data S1. Supporting Information. [file DOM-28-2859-s001.docx]

ESM A-1a Multiple Linear Regression Analysis for the MoCA

| Variable | β Coefficient (95% CI) | *P* value |
| --- | --- | --- |
| Intercept | 26.97 (24.32 to 29.61) | < 0.0001^*^ |
| FBG | -0.1224 (-0.2851 to 0.04028) | 0.1343 |
| HbA1c | 0.1506 (-0.03254 to 0.3336) | 0.1031 |
| Group  (T2DM-MCI vs. T2DM-nMCI) | -5.208 (-6.885 to -3.532) | < 0.0001^*^ |

Abbreviations: T2DM, type 2 diabetes mellitus; MCI, mild Cognitive Impairment; nMCI, without mild Cognitive Impairment; FBG, Fasting Blood Glucose; HbA1c, glycosylated hemoglobin; MoCA, Montreal Cognitive Assessment

^*^ Indicating *P* < 0.05 (applied Bonferroni correction)

ESM A-1b Multiple Linear Regression Analysis for the AVLT-H-D

| Variable | β Coefficient (95% CI) | *P* value |
| --- | --- | --- |
| Intercept | 5.340 (2.212 to 8.467) | 0.002^*^ |
| FBG | -0.050 (-0.2420 to 0.1427) | 0.601 |
| HbA1c | 0.108 (-0.108 to 0.325) | 0.314 |
| Group  (T2DM-MCI vs. T2DM-nMCI) | -1.362 ( -3.344 to 0.6193) | 0.170 |

Abbreviations: T2DM, type 2 diabetes mellitus; MCI, mild Cognitive Impairment; nMCI, without mild Cognitive Impairment; FBG, Fasting Blood Glucose; HbA1c, glycosylated hemoglobin; AVLT-H-D, Auditory Verbal Learning Test-Huashan version-delayed recall

^*^ Indicating *P* < 0.05 (applied Bonferroni correction)

ESM A-1c Multiple Linear Regression Analysis for the WMS-DST-T

| Variable | β Coefficient (95% CI) | *P* value |
| --- | --- | --- |
| Intercept | 13.77 (10.08 to 17.46) | < 0.0001^*^ |
| FBG | -0.1452 (-0.3720 to 0.08171) | 0.2003 |
| HbA1c | 0.3014 (0.04607 to 0.5567) | 0.0224^*^ |
| Group  (T2DM-MCI vs. T2DM-nMCI) | -2.470 (-4.807 to -0.1321) | 0.0392^*^ |

Abbreviations: T2DM, type 2 diabetes mellitus; MCI, mild Cognitive Impairment; nMCI, without mild Cognitive Impairment; FBG, Fasting Blood Glucose; HbA1c, glycosylated hemoglobin; WMS-DST-T, Wechsler Memory-Scale-digit Span Test-Total score

^*^ Indicating *P* < 0.05 (applied Bonferroni correction)

ESM A-1d Multiple Linear Regression Analysis for the WMS-DST-B

| Variable | β Coefficient (95% CI) | *P* value |
| --- | --- | --- |
| Intercept | 5.255 (3.483 to 7.027) | < 0.0001^*^ |
| FBG | -0.02813 (-0.1371 to 0.08084) | 0.6007 |
| HbA1c | 0.09382 (-0.02881 to 0.2165) | 0.1281 |
| Group  (T2DM-MCI vs. T2DM-nMCI) | -1.624 (-2.747 to -0.5014) | 0.0062^*^ |

Abbreviations: T2DM, type 2 diabetes mellitus; MCI, mild Cognitive Impairment; nMCI, without mild Cognitive Impairment; FBG, Fasting Blood Glucose; HbA1c, glycosylated hemoglobin; WMS-DST-B, Wechsler Memory Scale-Digit Span Test-Backward

^*^ Indicating *P* < 0.05 (applied Bonferroni correction)

ESM A-1e Multiple Linear Regression Analysis for the SVF

| Variable | β Coefficient (95% CI) | *P* value |
| --- | --- | --- |
| Intercept | 20.44 (15.51 to 25.37) | < 0.0001^*^ |
| FBG | 0.0004691 (-0.3027 to 0.3037) | 0.9975 |
| HbA1c | -0.01652 (-0.3577 to 0.3247) | 0.9216 |
| Group  (T2DM-MCI vs. T2DM-nMCI) | -3.708 (-6.832 to -0.5839) | 0.0218^*^ |

Abbreviations: T2DM, type 2 diabetes mellitus; MCI, mild Cognitive Impairment; nMCI, without mild Cognitive Impairment; FBG, Fasting Blood Glucose; HbA1c, glycosylated hemoglobin; SVF, Semantic Verbal Fluency

^*^ Indicating *P* < 0.05 (applied Bonferroni correction)

ESM A-1f Multiple Linear Regression Analysis for the APTw SI of Left Hippocampal Head

| Variable | β Coefficient (95% CI) | *P* value |
| --- | --- | --- |
| Intercept | 2.198 (0.9644 to 3.431) | 0.0011* |
| FBG | -0.0265 (-0.1023 to 0.04935) | 0.4796 |
| HbA1c | -0.0070 (-0.09231 to 0.07841) | 0.8686 |
| Group  (T2DM-MCI vs. T2DM-nMCI) | -0.03195 (-0.8134 to 0.7495) | 0.9338 |

Abbreviations: T2DM, type 2 diabetes mellitus; MCI, mild Cognitive Impairment; nMCI, without mild Cognitive Impairment; FBG, Fasting Blood Glucose; HbA1c, glycosylated hemoglobin; APTw SI, amide proton transfer-weighted signal intensity

^*^ Indicating *P* < 0.05 (applied Bonferroni correction)

ESM A-1g Multiple Linear Regression Analysis for the APTw SI of Left Hippocampal Body

| Variable | β Coefficient (95% CI) | *P* value |
| --- | --- | --- |
| Intercept | 2.025 (1.098 to 2.952) | 0.0001* |
| FBG | 0.00135 (-0.06279 to 0.06550) | 0.9658 |
| HbA1c | -0.0232 (-0.08020 to 0.03379) | 0.4108 |
| Group  (T2DM-MCI vs. T2DM-nMCI) | -0.2913 (-0.8785 to 0.2959) | 0.3178 |

Abbreviations: T2DM, type 2 diabetes mellitus; MCI, mild Cognitive Impairment; nMCI, without mild Cognitive Impairment; FBG, Fasting Blood Glucose; HbA1c, glycosylated hemoglobin; APTw SI, amide proton transfer-weighted signal intensity

^*^ Indicating *P* < 0.05 (applied Bonferroni correction)

ESM A-1h Multiple Linear Regression Analysis for the APTw SI of Left Hippocampal Tail

| Variable | β Coefficient (95% CI) | *P* value |
| --- | --- | --- |
| Intercept | 1.635 (0.8066 to 2.463) | 0.0004* |
| FBG | -0.0294 (-0.08031 to 0.02156) | 0.2470 |
| HbA1c | -0.0209 (-0.08020 to 0.03379) | 0.4620 |
| Group  (T2DM-MCI vs. T2DM-nMCI) | 0.1144 (-0.4104 to 0.6392) | 0.6583 |

Abbreviations: T2DM, type 2 diabetes mellitus; MCI, mild Cognitive Impairment; nMCI, without mild Cognitive Impairment; FBG, Fasting Blood Glucose; HbA1c, glycosylated hemoglobin; APTw SI, amide proton transfer-weighted signal intensity

^*^ Indicating *P* < 0.05 (applied Bonferroni correction)

ESM A-1i Multiple Linear Regression Analysis for the APTw SI of Right Hippocampal Head

| Variable | β Coefficient (95% CI) | *P* value |
| --- | --- | --- |
| Intercept | 1.396 (0.1833 to 2.609) | 0.0256* |
| FBG | 0.05283 (-0.02175 to 0.1274) | 0.1576 |
| HbA1c | -0.0368 (-0.1207 to 0.04714) | 0.3764 |
| Group  (T2DM-MCI vs. T2DM-nMCI) | 0.4091 (-0.3593 to 1.178) | 0.2843 |

Abbreviations: T2DM, type 2 diabetes mellitus; MCI, mild Cognitive Impairment; nMCI, without mild Cognitive Impairment; FBG, Fasting Blood Glucose; HbA1c, glycosylated hemoglobin; APTw SI, amide proton transfer-weighted signal intensity

^*^ Indicating *P* < 0.05 (applied Bonferroni correction)

ESM A-1j Multiple Linear Regression Analysis for the APTw SI of Right Hippocampal Body

| Variable | β Coefficient (95% CI) | *P* value |
| --- | --- | --- |
| Intercept | 1.189 (0.3967 to 1.981) | 0.0047* |
| FBG | 0.0244 (-0.02437 to 0.07308) | 0.3141 |
| HbA1c | -0.0167(-0.07155 to 0.03812) | 0.5369 |
| Group  (T2DM-MCI vs. T2DM-nMCI) | 0.2646 (-0.2374 to 0.7667) | 0.2890 |

Abbreviations: T2DM, type 2 diabetes mellitus; MCI, mild Cognitive Impairment; nMCI, without mild Cognitive Impairment; FBG, Fasting Blood Glucose; HbA1c, glycosylated hemoglobin; APTw SI, amide proton transfer-weighted signal intensity

^*^ Indicating *P* < 0.05 (applied Bonferroni correction)

ESM A-1k Multiple Linear Regression Analysis for the APTw SI of Right Hippocampal Tail

| Variable | β Coefficient (95% CI) | *P* value |
| --- | --- | --- |
| Intercept | 1.808 (0.9752 to 2.640) | 0.0001* |
| FBG | 0.0103 (-0.04094 to 0.06147) | 0.6840 |
| HbA1c | -0.031 (-0.08866 to 0.02660) | 0.2790 |
| Group  (T2DM-MCI vs. T2DM-nMCI) | -0.2972 (-0.8248 to 0.2304) | 0.2578 |

Abbreviations: T2DM, type 2 diabetes mellitus; MCI, mild Cognitive Impairment; nMCI, without mild Cognitive Impairment; FBG, Fasting Blood Glucose; HbA1c, glycosylated hemoglobin; APTw SI, amide proton transfer-weighted signal intensity

^*^ Indicating *P* < 0.05 (applied Bonferroni correction)

ESM A-2 Correlation of neuropsychological variables and APTw signal intensity in the hippocampi of T2DM-nMCI patients

| Pearson  (r, *P*) | MoCA | TMT-A | SVF | WMS  -DST-F | WMS  -DST-B | WMS  -DST-T | AVLT-H-I | AVLT-H-D |
| --- | --- | --- | --- | --- | --- | --- | --- | --- |
| L-head  L-body  L-tail  R-head  R-body  R-tail | -0.115,0.787  0.386,0.346  -0.448,0.266  0.463,0.248  0.328,0.427  -0.077,0.857 | 0.258,0.537  0.140,0.741  0.025,0.953  -0.152,0.719  -0.473,0.236  -0.131,0.757 | 0.770,0.025^*^  0.293,0.482  0.249,0.552  -0.117,0.784  -0.020,0.962  0.244,0.561 | 0.459,0.253  -0.348,0.399  -0.008,0.985  -0.258,0.537  0.139,0.743  -0.159,0.708 | -0.802,0.017^*^  0.028,0.948  -0.275,0.510  -0.027,0.949  -0.182,0.666  0.008,0.985 | -0.702,0.053  -0.212,0.615  -0.358,0.384  -0.218,0.603  -0.135,0.750  -0.102,0.810 | 0.073,0.863  0.491,0.217  0.503,0.204  0.655,0.078  0.584,0.128  0.607,0.111 | -0.027,0.950  0.241,0.566  0.468,0.242  0.513,0.193  0.564,0.145  0.514,0.192 |

Abbreviations: T2DM, type 2 diabetes mellitus; MCI, mild Cognitive Impairment; APTw, amide proton transfer-weighted; L, left; R, right; MoCA, Montreal Cognitive Assessment; TMT-A, Trail-Making Test- part A; SVF, Semantic Verbal Fluency; WMS-DST-T, Wechsler Memory-Scale-digit Span Test-Total score; WMS-DST-F, Wechsler Memory Scale-Digit Span Test-Forward; WMS-DST-B, Wechsler Memory Scale-Digit Span Test-Backward; AVLT-H-I, Auditory Verbal Learning Test-Huashan version-immediate recall; AVLT-H-D, Auditory Verbal Learning Test-Huashan version-delayed recall

^*^ Indicating *P* < 0.05 (applied Bonferroni correction)

ESM A-3 Correlation of neuropsychological variables and APTw signal intensity in the hippocampi of T2DM-MCI patients

| Pearson  (r, *P*) | MoCA | TMT-A | SVF | WMS  -DST-F | WMS  -DST-B | WMS  -DST-T | AVLT-H-I | AVLT-H-D |
| --- | --- | --- | --- | --- | --- | --- | --- | --- |
| L-head  L-body  L-tail  R-head  R-body  R-tail | 0.071,0.742  -0.185,0.208  0.033,0.822  -0.025,0.909  0.207,0.159  0.054,0.716 | -0.105,0.624  0.031,0.834  -0.333,0.021^*^  0.186,0.385  -0.017,0.908  0.083,0.574 | 0.414,0.044^*^  0.188,0.200  0.141,0.170  -0.187,0.382  0.001,0.996  0.224,0.127 | 0.101,0.637  0.086,0.561  0.143,0.164  -0.129,0.548  -0.068,0.647  0.140,0.342 | -0.272,0.199  -0.161,0.275  0.113,0.274  0.057,0.792  -0.139,0.346  -0.136,0.357 | -0.098,0.650  -0.197,0.179  0.118,0.251  -0.041,0.848  -0.135,0.362  -0.012,0.940 | 0.079,0.714  0.280,0.054  0.013,0.903  -0.130,0.545  -0.275,0.059  -0.025,0.864 | -0.077,0.720  0.041,0.781  -0.376,0.000^*^  -0.249,0.241  -0.180,0.220  0.109, 0.612 |

Abbreviations: T2DM, type 2 diabetes mellitus; MCI, mild Cognitive Impairment; APTw, amide proton transfer-weighted; L, left; R, right; MoCA, Montreal Cognitive Assessment; TMT-A, Trail-Making Test- part A; SVF, Semantic Verbal Fluency; WMS-DST-T, Wechsler Memory-Scale-digit Span Test-Total score; WMS-DST-F, Wechsler Memory Scale-Digit Span Test-Forward; WMS-DST-B, Wechsler Memory Scale-Digit Span Test-Backward; AVLT-H-I, Auditory Verbal Learning Test-Huashan version-immediate recall; AVLT-H-D, Auditory Verbal Learning Test-Huashan version-delayed recall

^*^ Indicating *P* < 0.05 (applied Bonferroni correction)

ESM A-4 Diagnostic value of the APTw signal intensity in distinguishing T2DM-MCI from T2DM-nMCI in different regions of hippocampus

| Variable | L-head | L-body | L-tail | R-head | R-body | R-tail |
| --- | --- | --- | --- | --- | --- | --- |
| AUC  95% CI  *P* | 0.531  0.2759- 0.7870  0.802 | 0.651  0.4121- 0.8908  0.227 | 0.623  0.3944- 0.8513  0.327 | 0.617  0.3789- 0.8554  0.350 | 0.680  0.4902- 0.8698  0.151 | 0.637  0.3641-  0.9097  0.277 |

Abbreviations: T2DM, type 2 diabetes mellitus; MCI, mild Cognitive Impairment; nMCI, without mild Cognitive Impairment; AUC, Area under the curve; CI, Confidence interval; L, left; R, right

^*^ Indicating *P* < 0.05

Electronic Supplementary Material (ESM) -B


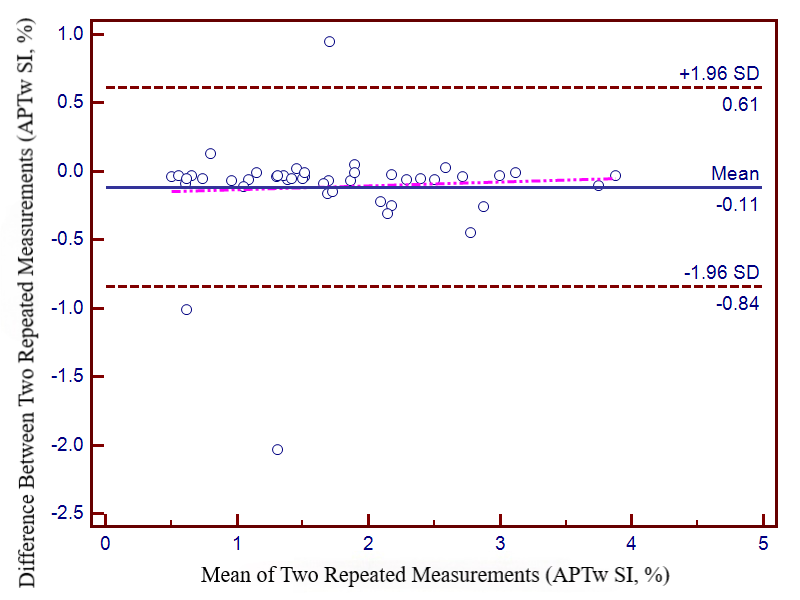


**Electronic Supplementary Material Legends**

**Electronic Supplementary Material (ESM) -A-1(a-k):** Multiple linear regression analysis for cognitive scores and APTw signal of hippocampus among T2DM-MCI and T2DM-nMCI

**Electronic Supplementary Material (ESM) -A-2:** Correlation of T2DM-nMCI group between neuropsychological variables and APTw signal intensity in various parts of bilateral hippocampus

**Electronic Supplementary Material (ESM) -A-3:** Correlation of T2DM-MCI group between neuropsychological variables and APTw signal intensity in various parts of bilateral hippocampus

**Electronic Supplementary Material (ESM) -A-4:** Diagnostic value of the APTw signal intensity in distinguishing T2DM-MCI from T2DM-nMCI in different regions of hippocampus

**Electronic Supplementary Material (ESM) -B:** Bland-Altman plots show APTw signal intensity values of APTw (%) between two repeated measurements.
